# Glucose metabolic dysregulation of diabetes and Its role in atrial fibrillation pathogenesis

**DOI:** 10.3389/fcvm.2025.1711069

**Published:** 2026-02-19

**Authors:** Yuqi Chen, Min Xu, Ke Wei, Nan Ma

**Affiliations:** Department of Cardiothoracic Surgery, Xinhua Hospital, School of Medicine, Shanghai Jiaotong University, Shanghai, China

**Keywords:** arrhythmia mechanisms, atrial fibrillation, diabetes mellitus, glucose metabolic dysregulation, metabolic remodeling

## Abstract

Atrial fibrillation (AF) is the most prevalent persistent arrhythmia in clinical practice, with a complex pathogenesis that remains incompletely understood. Emerging evidence underscores a strong association between diabetes and the occurrence of AF, highlighting a significantly elevated risk among diabetic patients. This increased susceptibility is not solely attributable to chronic hyperglycemia but is also shaped by blood glucose fluctuations and dysregulated glucose metabolism. In this review, we summarize the mechanisms by which hyperglycemia and blood glycemic variability contribute to the onset of AF, and the potential involvement of abnormal glucose metabolism pathways and metabolic disturbances induced by diabetes in the pathogenesis of AF.

## 1 Introduction

AF is the most prevalent arrhythmia in clinical practice. According to the 2019 Global Burden of Disease (GBD) study data, approximately 59.7 million people worldwide suffer from AF (1), making a significant increase from the 45.6 million cases reported in 2010 (2). This rising prevalence indicates that AF and its associated complications (including stroke, heart failure, myocardial infarction, cardiovascular death, and dementia) will persistently exacerbate the burden on healthcare systems (3, 4). Despite significant efforts to investigate the pathophysiology of AF and develop improved treatment methods in recent years, due to the complex pathogenesis of AF and significant interindividual variability, it is still difficult to determine the specific causes in each patient, and clinical efficacy needs to be further improved (5). Recent research has revealed that patients with diabetes face a heightened risk of AF, suggesting a potential association between diabetes and AF risk factors (5–8). This association may be driven by disturbances in blood glucose levels and glucose metabolism, which can influence the onset and progression of AF. Consequently, this review will focus on the primary characteristics of diabetes, exploring the potential pathophysiological connections between hyperglycemia, abnormal glucose metabolism, and AF.

## 2 Epidemiology of atrial fibrillation and diabetes

Many epidemiological studies have suggested a close relationship between diabetes and AF. In the landmark Framingham Heart Study, the incidence rate of AF in patients with diabetes was significantly higher than that in non-diabetic patients, with a 1.4-fold and 1.6-fold increase in risk for men and women, respectively (6). In the VALUE trial, a follow-up of more than 4 years in 15,245 patients revealed an AF incidence of 5.4% in those with diabetes, compared with 3.8% in non-diabetic patients. Diabetes was associated with a 49% increased risk of new-onset AF compared with non-diabetic patients (7). In the ARIC study, a total of 13,025 participants were divided into diabetes groups based on HbA1c: no diabetes HbA1c < 5.7%, pre-diabetes HbA1c 5.7%–6.4%, and diabetes HbA1c > 6.5%. Over 20 years of follow-up, the incidence of AF was 9.02 per 1,000 person-years in diabetic patients, compared with 4.51 per 1,000 person-years in non-diabetic patients, indicating that AF incidence in diabetic patients was twice that of non-diabetic patients. Moreover, the incidence of AF increased to 5.14 per 1,000 person-years in pre-diabetes (8). The impact of diabetes on AF is also reflected in the recurrence following catheter ablation. After an average follow-up of 18 months, 2,504 patients who underwent AF catheter ablation had a 32% recurrence rate among those with diabetes, compared with 25.3% in non-diabetic patients—a statistically significant difference (9). These results suggest that diabetes may increase susceptibility to AF and contributes to higher recurrence rates following intervention.

Abnormal blood glucose levels are a hallmark of diabetes. Hyperglycemia, a common feature in diabetic patients, is closely linked to the onset of atrial fibrillation. In a meta-analysis, Aune et al. demonstrated that hyperglycemia correlates positively with AF occurrence and identified a dose-response relationship between elevated blood glucose levels and AF risk. Specifically, for every 20 mg/dL increase in blood glucose, the relative risk of AF rises by 11% (10). A large-scale Swedish cohort study categorized participants into five groups based on HbA1c levels: ≤6.9%, 7.0%–7.8%, 7.9%–8.7%, 8.8%–9.6%, and ≥9.7%. After a mean follow-up of 5 years, the risk of developing AF in these groups was 24%, 28%, 38%, 51%, and 55% compared to the general population, respectively (11). The findings indicate a significant increase in the risk of AF with rising blood glucose levels. Furthermore, hyperglycemia may influence AF recurrence. In a follow-up study involving 228 patients with paroxysmal AF who underwent catheter ablation, the recurrence rate among diabetic patients with fasting blood glucose levels of 129 ± 37 mg/dL was 18.5%, notably higher than the 8% recurrence rate in the patients with fasting blood glucose levels of 85 ± 15 mg/dL (12).

Glycemic fluctuations are another common feature among patients with diabetes. Several epidemiological studies have found that blood glucose fluctuations is associated with an increased risk of AF. A retrospective cohort study with a median follow-up of 6.9 years found that patients with high glycemic variability had a significantly elevated risk of new-onset AF, and statistical analysis confirmed that glycemic variability was an independent risk factor for AF (13). In addition, a cohort study in Taiwan involving 27,246 patients with type 2 diabetes showed that patients with greater blood glucose variability had a 29% higher risk of developing AF compared to the control group (14).

Abnormal blood glucose levels alone do not appear to account for the increased incidence of AF in diabetes, as even patients with well-controlled blood glucose levels exhibit a significantly higher risk of AF. Findings from the Swedish cohort study indicate that even among diabetic patients with well-controlled blood glucose (HbA1c ≤ 6.9%), the incidence of AF remains 24% higher than in the general population (11). Similar trends have been observed in other epidemiological studies. A Polish study involving 30-day ECG monitoring of 811 diabetic patients with AF aged >65 years found no significant difference in AF incidence between those with poor glycaemic control (HbA1c ≥ 7.5%) and those with strict glycemic control (HbA1c ≤ 6.5%)—77.1% vs. 83.6% (*p* > 0.3) (15). Although blood glucose control influences AF occurrence to some extent, it is not the sole determinant, as demonstrated in the ACCORD study. In this trial, 10,082 patients with type 2 diabetes were divided into two groups: intensive glycemic control (target HbA1c < 6.0%) and standard control (target HbA1c 7.0%–7.9%). The annual incidence of new-onset AF showed no significant difference between the groups, at 5.9 vs. 6.37 cases per 1,000 person-years (16). These findings suggest that abnormal glucose metabolism precedes hyperglycemia and serves as an independent risk factor for atrial fibrillation, distinct from hyperglycemia itself.

Findings from the aforementioned epidemiological studies indicate a strong association between diabetes and AF. While blood glucose levels significantly influence AF onset, they are not the sole contributing factor. Dysregulated glucose metabolism also plays a key role. In the following sections, we review common changes in glucose levels and metabolism in diabetes to explore the pathophysiological relationship between AF and diabetes. The key characteristics and findings of these pivotal epidemiological studies are summarized in Table 1.

**Table 1:** Epidemiological studies of diabetes and atrial fibrillation risk.

First Author (Year)	Study Name/Cohort	Study Design	Study Population & Country	Follow-up Duration	Diabetes/Glycemic Status Assessment	AF Identification Method	Key Findings (Effect Size, 95% CI)	Main Contribution/Conclusion
Benjamin et al. (6)	Framingham Heart Study	Prospective cohort study	*n* = 4,731 (2,090 male, 2,641 female), 55–94 years, US community population	Up to 38 years	Fasting glucose ≥7.77 mmol/L (≥140 mg/dL), or random glucose ≥11.11 mmol/L (≥200 mg/dL), or using insulin/oral hypoglycemic agents	ECG reviewed by study cardiologists (including routine study exams, hospital records, and external physician records)	Diabetes vs. no diabetes (multivariate adjusted):	This landmark study first identified diabetes as an independent risk factor for new-onset AF in both men and women in a large, community-based cohort, independent of age, hypertension, heart failure, and other factors. Also quantified the population attributable risk of diabetes for AF.
							• Men: OR 1.4 (*P* < .05) • Women: OR 1.6 (*P* < .05)	
Aksnes et al. (7)	VALUE Trial	Post-hoc analysis of RCT	*N* = 13,760 (hypertensive patients at high risk without AF at baseline), from 31 countries	Mean 4.2 years	Per WHO criteria: using glucose-lowering drugs, or fasting glucose ≥7.0 mmol/L (126 mg/dL). New-onset diabetes determined during follow-up.	12-lead ECG analyzed by central lab (at baseline and annually)	New-onset diabetes vs. always no diabetes:	Confirmed in high-risk hypertensive patients that new-onset diabetes is significantly associated with increased AF risk, suggesting early-stage glycemic dysregulation has important impact on AF development.
							• New-onset AF: HR 1.49 (1.14–1.94) • Persistent AF: HR 1.87 (1.28–2.74)	
Huxley et al. (8)	ARIC Study	Prospective cohort study	*n* = 13,025, 45–64 years, US community population (including White and African American)	Mean 14.5 years	Per ADA criteria: fasting glucose, HbA1c, medication history, or physician diagnosis	Study visit ECG, hospital ICD codes, and death certificates	Diagnosed diabetes vs. no diabetes: HR 1.35 (1.14–1.60)	Revealed independent positive association between diabetes, high HbA1c levels (representing poor long-term glycemic control) and AF risk. Suggested dose-response effect of diabetes on AF risk, related to disease severity and duration.
							HbA1c (diabetic patients): Each 1% increase, AF risk HR 1.13 (1.07–1.20)	
Creta et al. (9)	European Multicentre Ablation Cohort	Multicenter observational study	*n* = 2,504 (234 diabetic patients), patients undergoing AF catheter ablation, from 7 European centers	Median 17 months	Clinical diagnosis	12-lead ECG and 24-hour Holter monitoring	Diabetic vs. non-diabetic patients post-ablation:	Extended diabetes-AF association to treatment domain, demonstrating diabetes is an independent risk factor for AF recurrence after catheter ablation, particularly in persistent AF patients. Suggests diabetes may cause more severe atrial myopathy, making sinus rhythm maintenance via local ablation more challenging.
							• 12-month AF recurrence rate: 32.0% vs. 25.3% (*p* = 0.031) • AF recurrence risk (multivariate adjusted): HR 1.39 (1.07–1.88)	
Aune et al. (10)	Systematic Review & Meta-analysis	Systematic review and meta-analysis	31 prospective cohort studies, pooled >10,244,043 participants	Not applicable	Definitions from original studies (including diabetes diagnosis, prediabetes, fasting glucose levels)	Methods from original studies (including ECG, hospital codes, etc.)	Diabetes vs. no diabetes: Pooled RR 1.28 (1.22–1.35)	This comprehensive meta-analysis provides highest-level summary evidence that diabetes and elevated glucose are important risk factors for AF. Quantified risk and revealed continuous dose-response relationship, greatly consolidating knowledge in this field.
							Fasting glucose: Each 20 mg/dL increase, AF risk RR 1.11 (1.04–1.18)	
							Linear dose-response relationship exists	
Seyed Ahmadi et al. (11)	Swedish National Diabetes Register (NDR) Cohort	Nationwide cohort study	421,855 type 2 diabetes patients vs. 2,131,223 matched general population controls, Sweden	Median 5.0 years	Stratified by time-updated mean HbA1c levels	Diagnostic codes in National Patient Register	Type 2 diabetes vs. matched controls (fully adjusted): HR 1.28 (1.26–1.30)	Confirmed type 2 diabetes is an independent risk factor for AF and quantified risk at different glycemic control levels, showing AF risk persists even in well-controlled patients.
							Risk increases with HbA1c elevation. Even with HbA1c ≤ 6.9%, risk remains elevated (HR 1.16, 1.14–1.19)	
Chao et al. (12)	Not applicable (single-center cohort)	Observational cohort study	228 symptomatic, drug-refractory paroxysmal AF patients undergoing first catheter ablation, Taiwan, China	Mean 18.8 ± 6.4 months	Per ADA standards, defined as diabetes or impaired fasting glucose	AF/atrial tachycardia episodes >1 min recorded by ECG, Holter post-ablation	1. Baseline glucose: Dysglycemia group fasting glucose 119 ± 28 mg/dL (diabetes subgroup 129 ± 37 mg/dL), normal group 87 ± 7 mg/dL. 2. Recurrence risk: Dysglycemia is independent risk factor for post-ablation AF recurrence: Adjusted HR = 3.247 (1.209–8.720).	First to demonstrate in humans that dysglycemia (including prediabetes) worsens atrial electrophysiologic substrate and significantly increases AF recurrence risk post-ablation, providing key mechanistic explanation and prognostic evidence.
Gu et al. (13)	Single-center retrospective cohort	Am	505 type 2 diabetes patients without AF history, China	Median 6.9 years	At least 4 HbA1c measurements, long-term glycemic variability assessed by standard deviation (HbA1c-SD)	Diagnosed by standard ECG or Holter during follow-up	Higher glycemic variability is significant predictor of new-onset AF:	First to reveal in type 2 diabetes patients that long-term glycemic variability (not just average glucose levels) is an independent risk factor for new-onset AF, providing new management target for AF prevention.
							HbA1c-SD: HR = 1.726 (1.251–2.381), *p* = 0.001	
Hsu et al. (14)	NTUH-iMD Cohort (Taiwan University Hospital Integrated Medical Database)	Retrospective cohort study	27,246 type 2 diabetes patients (without AF, heart failure, severe renal disease history), Taiwan, China	Median 70.7 months (approximately 5.9 years)	Visit-to-visit glycemic variability, assessed by HbA1c Variability Score (HVS)	Diagnostic codes in electronic health records or standard 12-lead ECG	Highest HVS quartile significantly associated with new-onset AF risk:	Confirmed in large diabetes cohort that higher long-term visit-to-visit HbA1c variability (HVS) is an independent predictor of new-onset AF, emphasizing importance of stable long-term glucose for AF prevention.
							HVS Q4 vs. Q1: HR = 1.29 (1.12–1.50), *p* < 0.001	
Gumprecht et al. (15)	NOMED-AF Study	Cross-sectional observational study	Poland, *n* = 881 (≥65 years, with diabetes)	Not applicable (cross-sectional)	HbA1c, diabetes duration, glucose-lowering treatment regimen	Long-term ECG monitoring via remote monitoring vest for average 21.9 ± 9.1 days	AF prevalence: No significant association between diabetes duration, treatment regimen, or HbA1c levels and AF prevalence.	In Polish diabetic population ≥65 years, diabetes severity, treatment regimen, or glycemic control itself was not associated with AF prevalence. Independent predictors of AF were age, BMI, and low LDL, while strict glycemic control (HbA1c ≤ 6.5) was associated with lower risk of silent AF.
							Silent AF independent predictor: HbA1c ≤ 6.5 (OR 0.46, 95%CI 0.25–0.85).	
Fatemi et al. (16)	ACCORD Trial	Randomized controlled trial	US and Canada, *n* = 10,082 (type 2 diabetes, high risk)	Median 4.68 years	HbA1c (intensive group target <6.0%, standard group target 7.0%–7.9%)	12-lead ECG at study start, every two years, and end	AF incidence: Intensive vs. standard: 5.9/1,000 person-years vs. 6.37/1,000 person-years (*p* = 0.52).	In high-risk type 2 diabetes patients, intensive glycemic control did not reduce new-onset AF incidence compared to standard therapy. However, once AF occurred, patients had significantly increased risks of death, MI, and HF.
							New-onset AF significantly associated with increased risk of all-cause death, MI, and HF.	

## 3 Blood glucose levels and AF

### 3.1 Hyperglycemia and AF

Hyperglycemia caused by insulin resistance is a common phenomenon in type 2 diabetes. Insulin resistance, characterized by reduced sensitivity of cells to insulin, impairs the hormone's ability to promote glucose absorption and utilization (17). On one hand, impaired insulin signaling inhibits the translocation of glucose transporters (GLUT) to the cell membrane, limiting cellular glucose uptake (18). On the other hand, in insulin resistance, the heart adapts metabolically by increasing fatty acid utilization and reducing glucose utilization to meet its high energy demands (19). This adaptation further obstructs glucose utilization by the heart.

Hyperglycemia can directly induce AF. It triggers endoplasmic reticulum stress in cardiomyocytes. Yuan et al. demonstrated that high blood glucose upregulates mitochondrial fusion protein 2 (Mfn2), promoting endoplasmic reticulum stress, mitochondrial dysfunction mediated by Ca^2+^ overload, and subsequent cardiomyocyte death (20). Hyperglycemia also stimulates mast cells, contributing to AF. In a diabetic mouse model, high blood glucose increased cardiac fibrosis and reduced resting heart rate (21), predisposing the mice to AF. These effects were attenuated in the absence of mast cells (22). Additionally, hyperglycemia affects the heart by altering red blood cell secretion, by inducing diabetic patients to release more extracellular vesicles (EVs) containing phosphatidylserine, which may impair heart function by causing endothelial cell dysfunction (23). In diabetic patients with AF, EV secretion is elevated (24), suggesting a significant role for EVs in diabetes-related AF.

### 3.2 Blood glucose fluctuation and AF

Hyperglycemia can induce AF, and fluctuations in blood glucose levels due to poor control can further increase AF incidence. Glycemic variability (GV) assesses the magnitude and stability of blood glucose fluctuations. Currently, international consensus recommends the coefficient of variation (CV) as the core metric, calculated from short-term (typically 24-hour) continuous glucose monitoring data, reflecting the relative amplitude of glucose fluctuations around the mean. CV < 36% is defined as low GV (stable glucose), while CV ≥ 36% indicates high GV (unstable glucose), which is independently associated with increased oxidative stress and cardiovascular risk (25). In research settings, GV is often defined by quartiles of long-term metrics (such as fasting glucose coefficient of variation, FGCV, or HbA1c variability score, HVS). For instance, in the study by Hsu et al. (14), high GV was specifically defined as a fasting glucose CV > 26.2% or an HVS > 51.1%. Both short-term and long-term GV are influenced by factors such as diet, exercise, and medication adherence (26, 27).

Blood glucose fluctuations primarily induce AF by exacerbating cardiac fibrosis, activating oxidative stress, and damaging the autonomic nervous system. In a diabetic rat model, Saito et al. induced blood glucose fluctuations through controlled diet and additional insulin injections. They observed significantly increased myocardial fibrosis and higher AF susceptibility in rats with fluctuating glucose levels compared to controls, indicating that higher GV promotes AF through cardiac fibrosis (28).

High GV levels are also linked to increased oxidative stress. Clinical studies have shown that reactive oxygen metabolite levels in type 2 diabetes patients rise in sync with GV (29). Intermittent high glucose levels stimulate excessive reactive oxygen species (ROS) production and cell apoptosis more than stable high glucose environments (30). Animal studies revealed that blood glucose fluctuations promote ROS production by altering the AKT signaling pathway and upregulating Txnip expression, leading to increased cardiac fibrosis and prolonged action potential conduction, both of which promote AF (28).

GV also affects autonomic nervous function, which plays a critical role in AF initiation and maintenance (31). Xu et al. found that diabetic patients with high GV are at greater risk of autonomic neuropathy (32). Kalopita et al. demonstrated that high GV inhibits vagal nerve function in diabetic patients, leading to a relative dominance of the sympathetic nervous system and consequently increasing heart rate (33). It is important to note that this GV-induced tachycardia (fast heart rate) due to autonomic imbalance presents a different pathophysiology from the bradycardia (slow heart rate) observed in advanced diabetic models, which is primarily caused by structural damage to the sinoatrial node, such as fibrosis and apoptosis induced by persistent hyperglycemia and oxidative stress (21, 33). These findings collectively suggest that GV-mediated autonomic nervous changes contribute to AF development through a mechanism distinct from the structural remodeling seen in chronic hyperglycemia.

## 4 Glucose metabolism in diabetes and AF

In addition to the direct impact of blood glucose on AF, glucose metabolism disorders in diabetes also play a significant role in this process. Diabetes-induced disruptions in glucose transport and utilization alter normal glucose metabolism. It is widely accepted that glucose aerobic oxidation is reduced, while the activity of alternative metabolic pathways such as glycolysis, the polyol pathway, and the hexosamine biosynthesis pathway is significantly increased. These changes lead to the accumulation of metabolites, including glycogen and advanced glycation end products (AGEs). Such metabolic shifts not only cause energy metabolism disorders but also damage cardiac structural integrity, exacerbate electrophysiological dysfunction, and increase susceptibility to AF (34). Below, we explore the impact of diabetes on AF through the lens of glucose metabolism pathways and changes in metabolic products.

### 4.1 Reduction of glucose aerobic oxidation

Glucose undergoes glycolysis in cells to produce pyruvate, which, under aerobic conditions, enters mitochondria and is fully oxidized via the tricarboxylic acid (TCA) cycle and electron transport chain, releasing substantial energy. Under anaerobic conditions, pyruvate is reduced to lactate in the cytoplasm, generating minimal energy.

In normal cardiomyocytes, aerobic oxidation is a primary energy source for cardiac activity, essential for maintaining heart function. Inhibition of aerobic oxidation disrupts the heart's energy supply, potentially leading to arrhythmias such as AF. In diabetic patients, glucose aerobic oxidation is impaired in multiple ways. Insulin resistance reduces the activity of GLUT transporters and key glycolytic enzymes (e.g., hexokinase and phosphofructokinase), limiting glucose uptake into cells (18, 35, 36). Additionally, the expression of glycolysis-related proteins, such as *β*-enolase and glucose-6-phosphate isomerase, is downregulated in diabetic patients with AF (37), further restricting pyruvate production and limiting substrates for aerobic oxidation (38). Diabetes also inhibits the TCA cycle, reducing its flux and causing abnormal metabolite accumulation in cardiomyocytes (39, 40). These changes collectively indicate reduced glucose aerobic oxidation in diabetes, impairing energy conversion efficiency and leading to insufficient cardiac energy supply.

As a high-energy-demanding organ, the heart's energy deficiency not only affects contractile function but also alters cardiomyocyte electrophysiology, including ion channel activity and calcium homeostasis. Key ion channels such as ATP-sensitive potassium channels, Na+/K+ ATPase, and Ca2+ ATPase require adequate energy for normal function (18). Energy deficiency inhibits these channels, prolonging atrial action potentials and increasing membrane potential instability (41), thereby raising the risk of early repolarization and triggered activity—key electrophysiological foundations for AF. Energy deficiency also reduces mitochondrial Ca^2+^ uptake and increases Ca^2+^ leakage through Ryanodine receptor 2 (RyR2), further disrupting calcium homeostasis and increasing AF susceptibility (42, 43).

### 4.2 Changes in glycolysis

Research shows that glucose oxidation is limited, and anaerobic glycolysis is enhanced in diabetic patients. During AF, the heart experiences ischemia and hypoxia due to high-frequency excitation and contraction, further limiting glucose oxidation and mitochondrial function (44). As a compensatory mechanism, anaerobic glycolysis is activated to provide rapid energy to cardiomyocytes. This enhanced glycolysis resembles the Warburg effect in tumor cells (45), and its long-term activation may promote AF initiation and maintenance.

In an AF canine model, the expression of glycolysis-related regulatory factors, particularly pyruvate dehydrogenase kinase-4 (PDK4), was significantly upregulated in atrial myocytes (46). PDK4 upregulation inhibits the pyruvate dehydrogenase complex (PDH), limiting pyruvate entry into the TCA cycle and promoting its conversion to lactate (47). This metabolic shift increases lactate production, with elevated lactate levels in the right atrial appendage tissue of AF patients serving as key evidence for AF development. Excessive lactate enters mitochondria via monocarboxylate transporters, inducing oxidative stress and cardiomyocyte apoptosis (48). Lactate accumulation also damages RyR2, inhibiting Ca^2+^ release and disrupting calcium homeostasis, further exacerbating energy metabolism disorders and increasing arrhythmia risk. Additionally, PDK4 may induce AF by regulating matrix metalloproteinase activity and promoting cardiac remodeling (49).

### 4.3 Inhibition of pentose phosphate pathway

The pentose phosphate pathway (PPP), parallel to glycolysis, produces NADPH and ribose phosphate, playing a critical role in glucose metabolism and cellular antioxidant defense. Glucose-6-phosphate dehydrogenase (G6PDH), a key PPP enzyme, regulates glucose-6-phosphate influx into the pathway (50). In diabetes, G6PDH is inhibited in the heart, impairing PPP function and affecting cardiomyocyte activity.

While the role of PPP in AF remains unclear, it is implicated in cardiac diseases such as myocardial ischemia and heart failure. During ischemia-reperfusion, PPP inhibition is associated with reduced cardiomyocyte survival. Katare et al. demonstrated that decreased G6PDH activity in diabetic mice correlates with myocardial dysfunction induced by ischemia-reperfusion, suggesting G6PDH inhibition may regulate Akt activity, leading to cardiomyocyte apoptosis (51). PPP inhibition also disrupts redox balance, as G6PDH maintains cellular glutathione levels and prevents oxidative stress-induced cardiac damage (52). High blood glucose directly inhibits G6PDH and the Akt/Pim-1/Bcl-2 signaling pathway, promoting ROS and AGE accumulation, which contribute to diabetic cardiomyopathy and post-ischemic heart failure (53, 54). These findings highlight PPP's role in maintaining cardiomyocyte viability and protecting against oxidative stress, though its specific role in AF requires further investigation.

### 4.4 Activation of the hexosamine biosynthesis pathway

In hyperglycemia, the hexosamine biosynthesis pathway (HBP) is significantly activated. Glucose or fructose is converted to glucose-6-phosphate (Glc6P), then to glucosamine-6-phosphate (GlcN6P) by the rate-limiting enzyme glucosamine-6-phosphate synthase (GFAT), and finally to UDP-N-acetylglucosamine (UDP-GlcNAc) (55). As a key substrate for O-GlcNAc modification, increased UDP-GlcNAc levels enhance O-GlcNAc-modified proteins, regulating protein function, stability, and cellular metabolism (56). In diabetic patients, O-GlcNAc-modified protein levels are elevated in the atria, closely associated with AF (57).

Increased O-GlcNAc modification triggers cardiac electrical remodeling. Erickson et al. found that acute hyperglycemia-induced O-GlcNAc modification activates calmodulin-dependent protein kinase II (CaMKII), increasing calcium leakage and causing contractile dysfunction and AF. Hyperglycemia also increases O-GlcNAc modification of the Nav1.5 sodium channel, impairing its function and reducing rapid sodium currents, which can trigger arrhythmias (58). Mesubi et al. confirmed these findings in type 1 and type 2 diabetic mouse models, showing that reducing O-GlcNAc modification conferred resistance to AF (59). Thus, regulating HBP and O-GlcNAc modification may offer new insights into AF mechanisms and therapeutic strategies.

### 4.5 Activation of the polyol pathway

At normal glucose levels, most glucose is metabolized via glycolysis, with minimal entry into the polyol pathway. In hyperglycemia, hexokinase becomes saturated, and glucose flux into the polyol pathway increases, accounting for over 30% of total glucose metabolism (60, 61). In this pathway, aldose reductase (AR) reduces glucose to sorbitol using NADPH, and sorbitol dehydrogenase (SD) converts sorbitol to fructose, with NAD+ converted to NADH (62).

Polyol pathway activation is linked to cardiovascular diseases, including atherosclerosis and myocardial infarction (63). Overexpression of AR in diabetic mice accelerates atherosclerosis (64), while AR inhibition ameliorates coronary intimal thickening in diabetic dogs (65). In ischemia-reperfusion injury models, AR inhibitors reduce infarct size, demonstrating cardioprotective effects (66). Although research on the polyol pathway in arrhythmias is limited, we can speculate on its potential role in AF based on existing evidence.

Polyol pathway activation disrupts the NAD+/NADH redox balance, potentially activating oxidative stress and inhibiting glucose metabolism. High AR and SD activity in hyperglycemia promotes NAD+ conversion to NADH, leading to NADH accumulation and NAD+ deficiency (67, 68). Excessive NADH promotes ROS formation in mitochondrial complex I (69), triggering oxidative stress and impairing oxidative phosphorylation, reducing energy production (70). NAD+ deficiency also limits sirtuin pathway activity (71), inhibiting deacetylation of metabolic regulatory proteins and disrupting glycolysis (72, 73). As discussed earlier, energy deficiency and oxidative stress significantly impact ion channel function and tissue integrity (18, 74), contributing to AF. Hwang et al. showed that AR inhibitors improve cytosolic redox balance, enhance glycolysis, and increase ATP levels, maintaining sodium and calcium homeostasis in cardiomyocytes post-reperfusion (75).

AR activation also impairs antioxidant capacity by competing with glutathione (GSH) for NADPH, reducing GSH levels and weakening antioxidant defenses (76, 77). Sorbitol accumulation induces hyperosmotic stress, as its low membrane permeability leads to intracellular osmotic pressure increases (78, 79), causing cell damage and apoptosis (80). Hyperosmotic stress increases Ca^2+^ influx and mitochondrial depolarization, reducing ATP production and triggering apoptosis (81).

Fructose accumulation from the polyol pathway promotes glycosylation and ROS production. Fructose is metabolized into fructose-3-phosphate and 3-deoxyglucosone, potent non-enzymatic glycosylation agents that produce AGEs (62, 82). Fructose also activates the protein kinase C (PKC) pathway, stimulating NADPH-oxidase(NOX) and ROS production (76, 83), exacerbating oxidative stress and AGE accumulation (74).

The polyol pathway plays a critical role in cardiovascular diseases, and its pathological mechanisms provide valuable insights into AF development.

## 5 Changes in metabolites

Diabetes is associated with the abnormal accumulation of various metabolites, which exacerbate AF susceptibility by affecting cardiac structure and function.

### 5.1 Glycogen deposition

Diabetes can lead to glycogen accumulation in the heart. *In vitro* experiments, rat cardiomyocytes treated with high glucose showed a significant increase in glycogen content, approximately doubling compared to controls (84). In diabetic rat models, abundant glycogen particles were observed in the left atrial appendage and atrium, linked to impaired glucose uptake and utilization (85). Studies in diabetic mice revealed that increased phosphorylation of glycogen synthase and decreased AMPK activity promote glycogen accumulation, resulting in a 35%–50% increase in cardiac glycogen content compared to normal mice (86). These findings consistently indicate significant glycogen accumulation in the diabetic heart.

Excessive glycogen deposition disrupts normal cardiomyocyte electrophysiology. Animal studies suggest that glycogen accumulation may form mechanical barriers, impairing intercellular conduction and promoting AF (87). Embi et al. observed glycogen distribution in goat hearts and found that denser interstitial glycogen was associated with slower conduction velocity. They proposed that glycogen acts as a mechanical barrier, interfering with electrical signal transmission, causing uneven wavefront activation, and increasing AF susceptibility (88).

Glycogen accumulation is also linked to cardiomyocyte remodeling and myolysis. Ausma et al. demonstrated that AF progression in goat hearts is accompanied by significant glycogen accumulation, cardiomyocyte enlargement, and myofibril loss (myolysis), indicating a connection between glycogen deposition and structural changes in cardiomyocytes (89). Zhang et al. further showed that glycogen-induced myolysis promotes cardiac fibrosis. In atrial appendage sections from AF dogs, areas with significant glycogen accumulation exhibited both myolysis and extensive collagen deposition (87). Thus, glycogen deposition contributes to myolysis and fibrosis, driving atrial structural remodeling and influencing AF initiation and maintenance.

### 5.2 Accumulation of advanced glycation end products

AGEs are compounds formed through non-enzymatic reactions in prolonged high-glucose environments (90). Key AGEs, such as carboxymethyl lysine (CML), carboxyethyl lysine (CEL), and pentosidine, have drawn attention due to their pathological roles. Under normal conditions, AGE production and metabolism are balanced, maintaining physiological levels. However, diabetes accelerates AGE production, leading to excessive accumulation (91–93).

AGEs contribute to AF through mechanisms involving inflammation, oxidative stress, and cellular aging. Binding of AGEs to their receptor RAGE activates inflammatory responses, promoting inflammatory cell infiltration and cytokine release (94–96). AGEs-RAGE binding significantly enhances the NF-*κ*B signaling pathway (97), which in turn upregulates RAGE expression, creating a positive feedback loop (98, 99). This loop amplifies NF-*κ*B activation, exacerbating inflammation and potentially creating a pro-AF environment (100). Begieneman et al. found increased CML deposition in atrial tissue of AF patients, correlating with upregulated VCAM-1 expression and inflammatory cell accumulation (101). This suggests CML enhances VCAM-1-mediated recruitment of neutrophils, lymphocytes, and macrophages, releasing pro-inflammatory cytokines like IL-6 and TNF-α, which exacerbate atrial inflammation and promote AF.

Oxidative stress is another key mechanism of AGE-induced AF. Wong et al. reported that AGEs-RAGE binding activates NOX in neutrophils, triggering ROS release (102). ROS not only activate transcription factors like NF-*κ*B but also damage cellular proteins, increase RAGE expression, and promote further AGE production, creating a vicious cycle of oxidative stress. Excessive ROS cause lipid peroxidation, mitochondrial and DNA damage (70, 74), impairing cardiomyocyte function and ion channel activity (18, 103), thereby increasing AF risk. Animal and clinical studies have established links between AF, NOX activation, and oxidative stress (104, 105), though the specific mechanisms require further exploration.

Recent studies suggest AGEs promote cardiomyocyte senescence, contributing to AF. Zheng et al. found that AGEs-treated HL-1 cardiomyocytes exhibited increased G1 phase senescence and DNA content compared to controls. In diabetic mice, AGEs activated the p16/Rb pathway, promoting cardiomyocyte senescence. Senescent atrial cells show reduced L-type calcium current (ICa,L), transient outward potassium current (Ito), and ultra-rapid delayed rectifier potassium current (IKur), prolonging action potential duration and increasing AF susceptibility. Knocking down p16/Rb using RAGE antibodies or siRNA reversed these changes (106).

AGEs also impair intercellular junction function. Dufeys et al. demonstrated that AGEs reduce the expression of gap junction proteins Cx43 and Cx40 by inhibiting the AMPK pathway, disrupting electrical coupling between cardiomyocytes. In diabetic rats, AGEs exposure decreased Cx43 and Cx40 expression and slowed intercellular conduction. AMPK agonists restored gap junction protein expression and improved intercellular coupling (107).

Additionally, AGEs exacerbate atrial fibrosis. Studies show that AGEs increase connective tissue growth factor (CTGF) expression via RAGE-mediated signaling, promoting fibrosis (108). In diabetic rats, CTGF overexpression correlated with myocardial interstitial fibrosis (109), while AGEs cross-linking inhibitors reduced CTGF expression and alleviated fibrosis (110). These findings suggest that AGEs accumulation in diabetes upregulates CTGF, exacerbating fibrosis and increasing myocardial stiffness, thereby promoting AF.

## 6 Therapeutic implications

Although, as mentioned earlier, some large-scale clinical trials have shown that intensive glycemic control failed to significantly reduce the risk of new-onset AF (6, 10, 11)—indicating that hyperglycemia is not the sole determinant of diabetes-associated AF—this has shifted focus towards the pleiotropic, “off-target” effects of specific antidiabetic drugs and their direct intervention in the atrial metabolic substrate. Current evidence highlights the prominent role of sodium-glucose cotransporter 2 (SGLT2) inhibitors. A meta-analysis of cardiovascular outcome trials for SGLT2 inhibitors demonstrated a significant 24% reduction in the risk of new-onset AF in patients with diabetes (HR 0.76, 95% CI 0.65–0.90) (111). A further large cohort study confirmed that SGLT2 inhibitor use was associated with an 39% lower risk of AF compared to dipeptidyl peptidase-4 inhibitor use 39% (HR 0.61, 95% CI 0.50–0.73) (112). Glucagon-like peptide-1 receptor agonists (GLP-1 RAs) also show promising signals. A real-world study involving over 190,000 patients with type 2 diabetes reported that GLP-1 RA use was associated with an 18% risk reduction in AF (HR 0.82, 95% CI 0.70–0.96) (113). As a first-line antidiabetic therapy, metformin has also garnered research support for its anti-AF potential. A systematic review and meta-analysis including more than 4 million patients with type 2 diabetes indicated that metformin use was associated with a 15% reduction in the risk of new-onset atrial fibrillation (HR 0.85, 95% CI 0.76–0.94) (114). The antidiabetic strategy also impacts AF progression following successful catheter ablation. A recent meta-analysis indicates that the use of specific antihyperglycemic agents (such as GLP-1) following ablation is associated with a significant 45% reduction in the risk of AF recurrence (HR = 0.549, 95% CI 0.315–0.956) (115).

Based on the diabetic atrial metabolic remodeling mechanisms systematically detailed in this review, future intervention strategies targeting specific metabolic pathways present a differentiated research and development landscape. Regarding the core metabolic shift of suppressed glucose aerobic oxidation and enhanced glycolysis, modulating PDK4 activity to ameliorate energy metabolism imbalance represents a crucial direction (46–49). For the PPP and the HBP pathway, clinically available specific inhibitors are currently lacking. However, basic research indicates that resveratrol enhances glucose-6-phosphate dehydrogenase activity to boost the antioxidant capacity of the PPP (116), whereas benfotiamine activates transketolase to divert metabolic flux away from the HBP (117). Both have demonstrated potential in animal models for alleviating atrial oxidative stress and electrical remodeling, providing important clues for the development of a new generation of targeted drugs. It is noteworthy that the polyol pathway possesses a specific inhibitor, epalrestat, which is an aldose reductase inhibitor with a long-term clinical history in treating diabetic neuropathy and a well-established safety profile (118). Its mechanism of action involves inhibiting the overactivation of the polyol pathway, thereby reducing the abnormal accumulation of sorbitol and the subsequent generation of reactive oxygen species, concurrently decreasing fructose production (119). Although no studies have directly confirmed the effect of epalrestat on AF prevention or treatment, its defined mechanism is highly relevant to the pathophysiology of AF. Therefore, animal experiments could be conducted to validate its impact on atrial electrophysiology and structural remodeling, alongside epidemiological monitoring of AF incidence in these long-term users, to systematically evaluate the preventive effect of epalrestat on new-onset AF in diabetic patients. This research pathway would provide direct clinical evidence for metabolic pathway intervention strategies.

## 7 Limitations

Although the aforementioned studies have constructed a complex mechanistic network linking diabetes to AF, it is necessary to consider the inherent limitations of the current evidence when evaluating it. Firstly, a significant translational gap exists in the core mechanistic evidence. The current understanding of specific pathway functions (e.g., the polyol pathway, pentose phosphate pathway) is predominantly derived from animal models such as rodents and canines. While these models are essential, inherent differences in disease progression, atrial physiology, and metabolic context compared to humans limit the direct extrapolation of these findings to the clinic. Secondly, the associations between many aspects of disordered glucose metabolism and AF are still based on indirect evidence and rational speculation. A primary aim of this review is to systematize these potential pathways, thereby highlighting this promising area of inquiry; future work must employ well-designed interventional studies (encompassing both animal experiments and clinical research) that, in the context of controlling glycemia or specific metabolic nodes, directly verify the causal links between these metabolites and direct indices of AF, such as electrophysiological and structural remodeling parameters. Finally, the path from target validation to therapeutic realization remains unclear. Even if the efficacy of a specific target is confirmed in preclinical models, a major challenge remains in achieving precise intervention in atrial tissue without disrupting systemic metabolic homeostasis.

## 8 Conclusion

**Figure 1:**
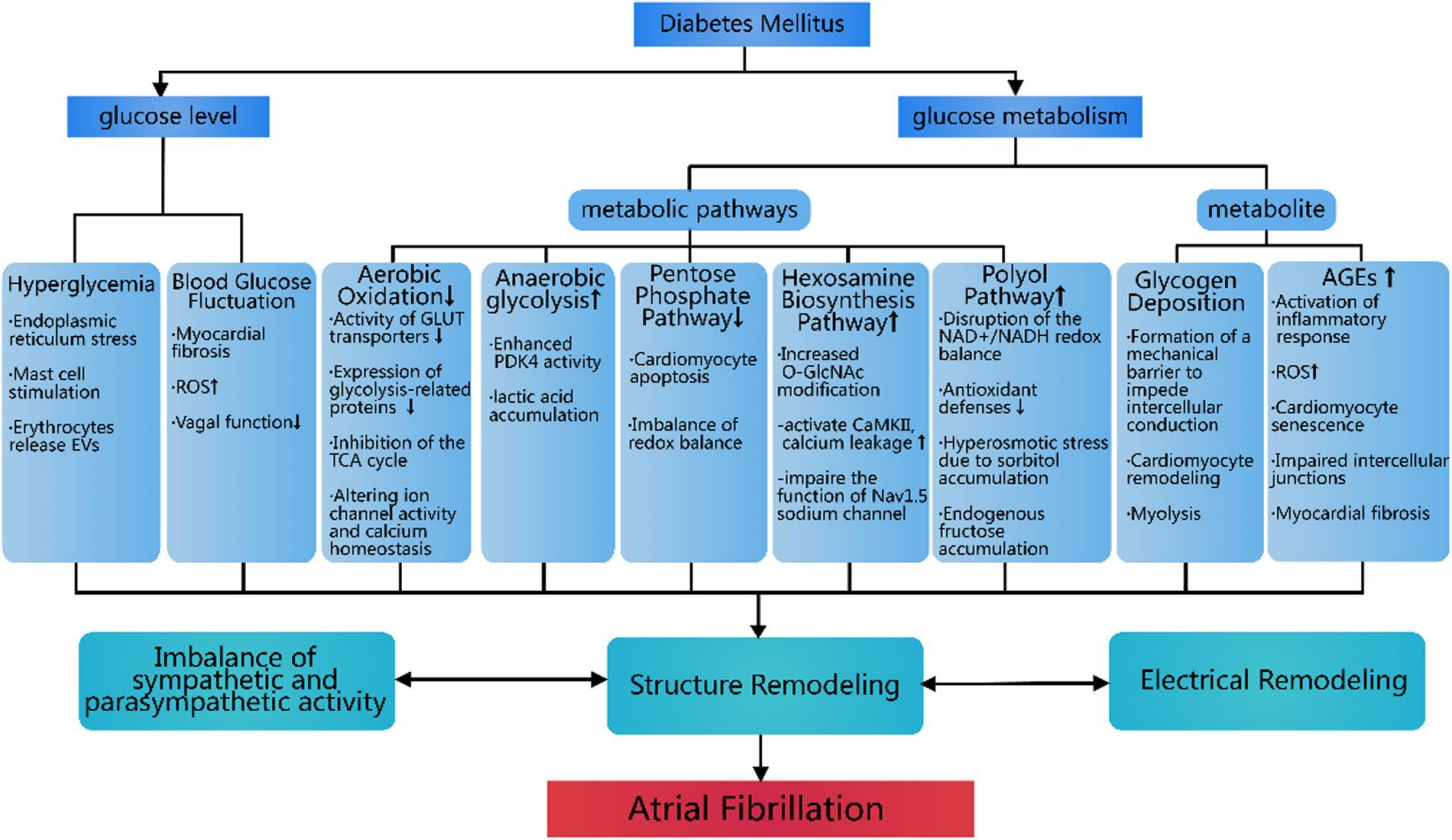
Metabolic disruptions in diabetes leading to atrial fibrillation. This figure systematically illustrates the core pathophysiological pathways through which diabetes mellitus promotes AF: 1) Dysregulated blood glucose levels (hyperglycemia and glycemic variability) serve as primary drivers, directly damaging atrial myocardium by inducing endoplasmic reticulum stress, activating oxidative stress (ROS), and causing autonomic imbalance; 2) Disordered glucose metabolic pathways (inhibition of aerobic oxidation, enhanced anaerobic glycolysis, activation of polyol and hexosamine pathways, and suppression of the pentose phosphate pathway) collectively lead to energy deficiency, calcium homeostasis disruption, and ion channel dysfunction; 3) Accumulation of detrimental metabolites (glycogen deposition and AGEs) ultimately contribute to atrial structural and electrical remodeling through the formation of conduction barriers, initiation of inflammation, and promotion of fibrosis, thereby establishing the pathological foundation for AF initiation and maintenance. EVs, Extracellular Vesicles; ROS, Reactive Oxygen Species; GLUT, Glucose Transporter; TCA, Tricarboxylic Acid; O-GIcNAc, O-linked N-acetylglucosamine; CaMKII, Calmodulin-Dependent Protein Kinase II.

Diabetes increases AF risk through hyperglycemia, blood glucose fluctuations, and glucose metabolism disorders and detrimental metabolites accumulation, leading to atrial structural remodeling and electrophysiological changes. The influence of glucose metabolic dysregulation for AF pathogenesis was summarized in Figure 1. While current research has elucidated some mechanisms linking diabetes and AF, many aspects remain unexplored. Future studies should focus on how diabetes-related metabolic pathways influence AF and investigate the potential of hypoglycemic drugs in AF prevention and treatment. Large-scale clinical trials are needed to validate these findings and provide evidence-based strategies for managing AF in diabetic patients.
